# Acceptance Rates of COVID-19 Vaccine Highlight the Need for Targeted Public Health Interventions

**DOI:** 10.3390/vaccines10081167

**Published:** 2022-07-22

**Authors:** Vered Shkalim Zemer, Zachi Grossman, Herman Avner Cohen, Moshe Hoshen, Maya Gerstein, Noga Yosef, Moriya Cohen, Shai Ashkenazi

**Affiliations:** 1 Dan-Petach Tikva District, Clalit Health Services, Petach Tikva 4972339, Israel; mosheho@clalit.org.il (M.H.); dpnoga@clalit.org.il (N.Y.); 2 Sackler Faculty of Medicine, Tel Aviv University, Tel Aviv 6997801, Israel; hermanc@tauex.tau.ac.il; 3 Adelson School of Medicine, Ariel University, Ariel 4070000, Israel; zgrosman@netvision.net.il (Z.G.); mayage@clalit.org.il (M.G.); shaias@ariel.ac.il (S.A.); 4 Maccabi Healthcare Services, Tel Aviv 6910107, Israel; 5 Pediatric Ambulatory Community Clinic, Petach Tikva 49504, Israel; 6 Bioinformatics Department, Jerusalem College of Technology, Jerusalem 9372115, Israel; 7 Microbiolog Unit, Ariel University, Ariel 4070000, Israel; moriya1cohen@gmail.com

**Keywords:** COVID-19, COVID-19 vaccine, adults, comorbidity, vaccine hesitancy

## Abstract

We aimed to examine rates of COVID-19 vaccination to elucidate the need for targeted public health interventions. We retrospectively reviewed the electronic medical files of all adults registered in a central district in Israel from 1 January 2021 to 31 March 2022. The population was characterized by vaccination status against COVID-19 and the number of doses received. Univariate and multivariable analyses were used to identify predictors of low vaccination rates that required targeted interventions. Of the 246,543 subjects included in the study, 207,911 (84.3%) were vaccinated. The minority groups of ultra-Orthodox Jews and Arabs had lower vaccination rates than the non-ultra-Orthodox Jews (68.7%, 80.5% and 87.7%, respectively, *p* < 0.001). Adults of low socioeconomic status (SES) had lower vaccination rates compared to those of high SES (74.4% vs. 90.8%, *p* < 0.001). Adults aged 20–59 years had a lower vaccination rate than those ≥60 years (80.0% vs. 92.1%, *p* < 0.0001). Multivariate analysis identified five independent variables that were significantly (*p* < 0.001) associated with low vaccination rates: minority groups of the ultra-Orthodox sector and Arab population, and underlying conditions of asthma, smoking and diabetes mellitus (odds ratios: 0.484, 0.453, 0.843, 0.901 and 0.929, respectively). Specific targeted public health interventions towards these subpopulations with significantly lower rates of vaccination are suggested.

## 1. Introduction

Coronavirus disease 2019 (COVID-19) was declared a global pandemic by the World Health Organization (WHO) on 11 March 2020 [1]. This pandemic has caused huge morbidity and mortality rates all over the globe; as of 15 July 2022, the WHO had recorded nearly 558 million confirmed cases of COVID-19, including 6.4 million deaths [2]. In addition to the acute illness, COVID-19 can also cause long-term complications and morbidity. The long-term illness of COVID-19 (referred to as long COVID or post-acute COVID-19) includes pulmonary, cardiovascular, neurological, hematological, multisystem inflammatory, renal, endocrine, gastrointestinal and integumentary sequelae [3].

Vaccination against the causative agent, the severe acute respiratory syndrome coronavirus 2 (SARS-CoV-2), is the leading strategy to control the COVID-19 pandemic worldwide [4]. A COVID-19 vaccine produced by Pfizer-BioNTech (BNT162b2) contains nucleoside-modified messenger RNA encoding the SARS-CoV-2 spike glycoprotein [5]. Two doses of this vaccine given to healthy adults 21 days apart elicited high neutralizing titers and robust, antigen-specific CD4+ and CD8+ T-cell responses against the virus [6,7]. BNT162b2 was 95% effective in preventing COVID-19 from 7 days after the second dose [8,9]. On 11 December 2020, the US Food and Drug Administration (FDA) authorized the Pfizer-BioNTech for COVID-19 prevention in persons 16 years of age or older [10].

Because of waning immunity and the development of SARS-CoV-2 mutants, on 30 July 2021, Israel was the first country to make a third dose (“booster dose”) of the BNT162b2 Pfizer vaccine available to all individuals aged ≥60 years who had been vaccinated at least 5 months earlier [11]. Later on, the booster program was extended to all the population aged 12 years or older [11].

A large observational study conducted by Barda et al., using nationwide mass vaccination data in Israel, showed that a third dose of the BNT162b2 mRNA COVID-19 vaccine is effective in preventing severe COVID-19-related outcomes. Adding a third vaccine dose was estimated as being 93% effective in preventing COVID-19-related hospitalization, 92% in preventing severe disease, and 81% in preventing COVID-19-related death, as of ≥7 days after the third dose [12].

The aim of our study was to explore factors associated with low acceptance of the COVID-19 vaccine in adults, in order to improve public health-targeted interventions in subpopulations with low vaccination rates. In this manuscript, we describe the study population and the methodology used and then report univariate and multivariate analyses of variables associated with vaccine acceptance.

## 2. Materials and Methods

Clalit Health Services (CHS) insures approximately 54% of the Israeli population. It maintains a comprehensive computerized database, continuously updated with regard to subjects’ demographics, community and outpatient visits, laboratory tests, hospitalizations, and medications prescribed and purchased. During each physician’s visit, a diagnosis is recorded according to the International Classification of Diseases, ninth revision (ICD-9).

The study population consisted of all subjects aged ≥20 years who were registered with CHS during the study period of 1 January 2021 to 31 March 2022 within the Dan-Petach Tikva administrative district. This district comprises about 500,000 members and includes large towns of mainly secular Jews, large towns of ultra-Orthodox Jews, and few Arab towns, with the majority being the non-ultra-Orthodox Jewish population. Israelis tend to live within neighborhoods based on this grouping.

The study population was divided into two groups: unvaccinated and vaccinated with at least one dose of the vaccine, with further subgroupings according to the number of COVID-19 vaccine doses received. The data collected from the electronic database included demographic information (age, gender, sector, and socioeconomic status (SES), which was defined according to the classification of the Israeli Central Bureau of Statistics [13]); vaccinations for COVID-19 (first, second and third dose) and for influenza in the previous three years; information regarding PCR testing (sampling dates and results); and comorbidities. The study was approved by the Clalit Community Institutional Review Board for Human Studies.

We extracted the study data into a central data table, which was anonymized for statistical analyses. Descriptive statistics were used to report the demographic and clinical variables of the vaccinated and unvaccinated study groups. Proportions were compared by a chi-square test or Fisher’s exact test, as appropriate, and continuous variables by Student’s t-test or a Mann–Whitney test, as appropriate. Variables associated with vaccine acceptance were first identified by univariate analysis. We then performed multivariate logistic regression to analyze the adjusted odds ratio of vaccination as the dependent variable, based on variables found significant in the univariate analysis. We drew vaccination uptake curves by sector adjusted for age and gender. We also drew a forest plot describing the adjusted relative association of the various variables on vaccine acceptance. Analysis was performed with R software (versions 4.1.0, Foundation for Statistical Computing, Vienna, Austria. URL https://www.R-project.org, accessed on 18 July 2022).

## 3. Results

Our study included all 246,542 adults aged ≥20 years from the Dan-Petach Tikva district. Of the study group, 125,819 (51%) were female and 120,724 (49%) males; 230,546 (93.5%) were Jews and 15,997 (6.5%) Arabs.

### 3.1. Factors Associated with COVID-19 Vaccine Acceptance

Table 1 presents the characteristics of our study population by receiving/not receiving the COVID-19 vaccine. Of the 256,543 adults in our study group, 207,911 (84.3%) received at least a single dose of the COVID-19 vaccine. Adults aged 20–59 years had a considerably lower vaccination rate than those aged ≥60 years (80.0% vs. 92.1%, *p* < 0.001). Males were less often vaccinated than females (83.6% vs. 85.0%, *p* < 0.001). The minority groups of ultra-Orthodox Jews and Arabs had lower vaccination rates than the non-ultra-Orthodox Jews (68.7%, 80.5%, and 87.7%, respectively, *p* < 0.001). Vaccination rates were also significantly related to SES: adults with low SES had lower vaccination rates compared to those with high SES (74.4% vs. 90.8%, *p* < 0.001). A higher COVID-19 vaccination rate was observed in adults who had received a previous seasonal influenza vaccine compared to those who did not (94.5% vs. 77.2%, *p* < 0.001).

Table 2 presents vaccination rates by underlying medical conditions. In general, the percentage of patients with underlying medical conditions was higher among the vaccinated than the unvaccinated population. Among medical conditions that were associated with increased risk of severe COVID-19, smoking, asthma, Down syndrome, depression, and obesity had relatively low rates among vaccinated individuals (82.7%, 85.4%, 87.2%, 87.5%, and 88.1%, respectively) as opposed to adults with cardiac disease, hypertension, cystic fibrosis, biological therapy or after solid organ transplantation, who had a high rate of >90% (92.4%, 92.5%, 92.9%, 94.7%, and 95.3%, respectively) (Table 2).

### 3.2. Analysis by Number of Vaccine Doses Received

Of the 207,911 adults who were vaccinated against COVID-19, 7.5% received only one single dose of the vaccine, 16.5% received two doses and 76% received the recommended three doses (Table 3).

The rates of receiving the first, second, and third doses of the COVID-19 vaccine were very similar between females and males. The number of vaccine doses received was significantly age-related: three doses of the vaccine were received by only 63% of individuals aged 20–39 years, compared to 74% and >80% in those aged 40–59 and >60 years, respectively. In comparison to the whole population, a higher number of vaccine doses were significantly given to individuals with high SES, non-ultra-Orthodox Jews, and those previously vaccinated against influenza (*p* < 0.001 for these three variables) (Table 3).

Table 4 presents the number of vaccine doses given to adults with various underlying medical conditions. The lowest rates of receiving the third COVID-19 vaccine dose were among adults with the following comorbidities: hematologic diseases (64.7%), steroid therapy (65.5%), smoking (75.8%), asthma (76.1%), neurologic diseases (76.5%), Down syndrome (77.1%) and obesity (78.5%).

**Figure 1:**
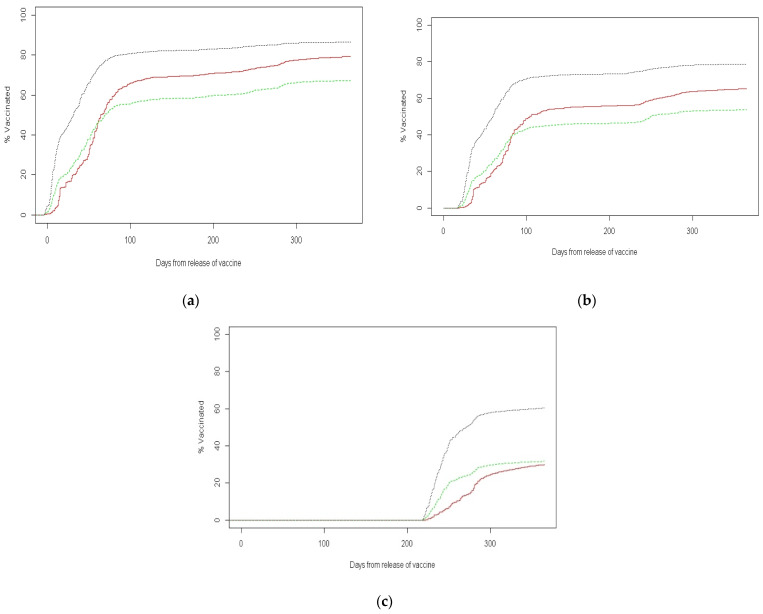
Cumulative rate with the time of vaccinated adults by sector. (**a**) First COVID-19 vaccine uptake; (**b**) second COVID-19 vaccine uptake; (**c**) third COVID-19 vaccine uptake.

Figure 1a–c shows the accumulated percentages of the vaccination with time by sector and according to the number of vaccine doses received. The figure refers to the time period from the approval of the vaccine dose for adults by the Israeli Ministry of Health until 31 March 2022. The vaccination uptake was slowest among ultra-Orthodox Jews, relatively slow in the Arab population, and fastest among non-ultra-Orthodox Jews.

### 3.3. Multivariate Analysis

Table 5 presents the results of the multivariate logistic regression analysis of the variables associated with COVID-19 vaccination rates among adults. The model identified five independent variables that were significantly (*p* < 0.001) associated with low vaccination rates. Among the demographic variables were the minority groups of ultra-Orthodox Jews and the Arab population (odds ratios: 0.484 and 0.453, respectively; *p* < 0.001 for both). Among the underlying conditions were individuals with asthma, smoking and diabetes mellitus (odds ratios: 0.843, 0.901 and 0.929, respectively; *p* values: <0.001, <0.001 and 0.011, respectively). In contrast, obesity was significantly and independently associated with an increased vaccine acceptance (odds ratio 1.086, *p* = 0.003).

**Figure 2:**
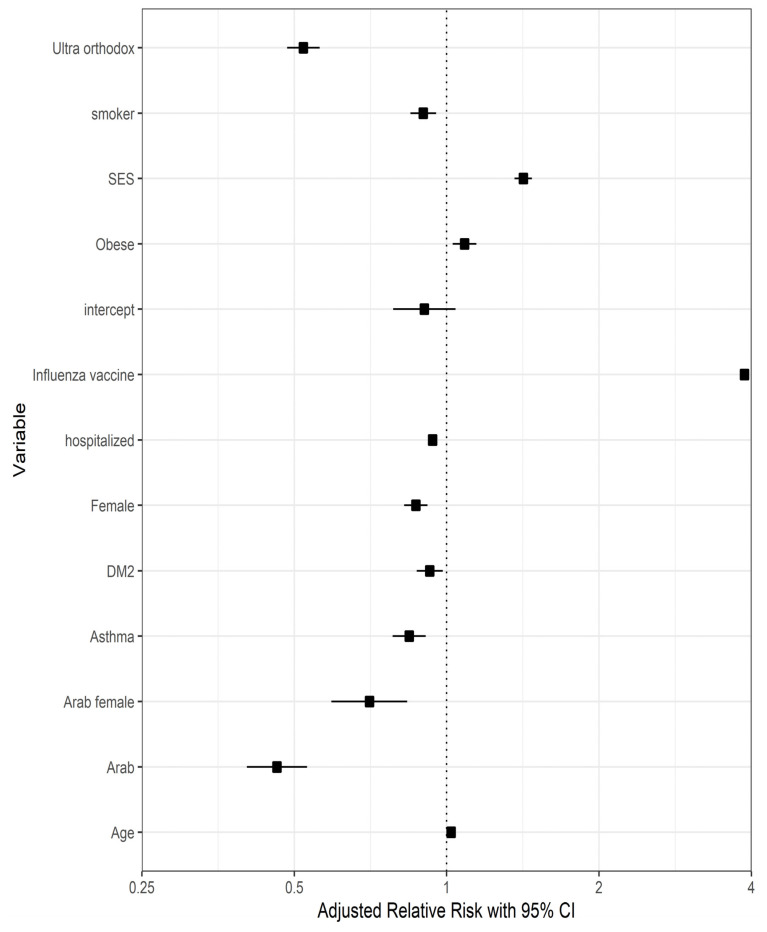
Forest plot describing the adjusted relative risks of various variables on vaccine acceptance.

Gender differences were noted among the minority groups: Arab women were at risk of a low vaccination rate, whereas ultra-Orthodox women showed a relatively high vaccination rate compared to corresponding males (odds ratios 0.728 and 1.136, respectively), although they were still less likely to be vaccinated than the non-ultra-Orthodox Jewish population. In the non-ultra-Orthodox Jewish population, after adjustment for age, elderly women were significantly less likely to vaccinate than men. Lower SES was negatively associated with getting vaccinated (odds ratio 0.705, *p* < 0.001). A non-parametric forest plot describing the adjusted relative risks of the various variables on vaccine acceptance is shown in Figure 2.

## 4. Discussion

In this large observational study, we explored factors associated with COVID-19 vaccination among Israeli adults to elucidate public health-targeted interventions that are needed to increase vaccine acceptance and reduce the COVID-19-associated morbidity and mortality. We found that Arabs and ultra-Orthodox Jews, which are two Israeli religious minority groups, had substantially lower vaccination rates compared to the non-ultra-Orthodox Jewish population.

The Israeli population consists of approximately 74% Jews, 21% Arabs, and 5% of other ethnicities [13]. The Arab population tends to live in crowded neighborhoods [14] and is at a relatively high risk of morbidity and mortality related to COVID-19 due to risk factors such as smoking, obesity, diabetes, hypertension, cardiovascular diseases, and lack of physical activity [15]. There are several potential impediments to COVID-19 vaccination among the Israeli Arab population, including difficulty in reaching vaccination sites, occasional language barriers, less exposure to the national media, concerns about potential adverse effects (especially on fertility and pregnancy), the perception that vaccination risks outweigh potential benefits, and unique needs and concerns of a culturally defined population group [16]. Focused, targeted public health measures are needed in this population, mostly through their trusted religious and community leaders and within their media, taking into account their special culture.

In the Jewish population, about 12% belong to a distinct subpopulation of the religiously ultra-Orthodox, with a distinct ultra-religious education system, often low SES, high fertility rates and crowded living conditions [17]. The ultra-Orthodox Jewish community in Israel is very closed-minded and has, by choice, reduced contact with the non-ultra-Orthodox Jewish population. Their religious leaders, the Rabbis, play a central role in their behavior, including around health issues and vaccine acceptance [18]. They have limited access to general media, and thus are prone to disinformation. Targeted public health interventions in this population are required based on their specific cultural principles, addressing their special concerns (i.e., fertility) and delivered through their Rabbinic and communal leaders, community newspapers, sectoral radio stations and other media [16].

Low SES groups have already been reported as disproportionately affected by the COVID-19 pandemic with higher morbidity and mortality rates, rendering high vaccination rates crucial in these groups [19,20,21]. Unfortunately, our study shows that adults with low SES had significantly lower COVID-19 vaccination rates. Our results are similar to those reported by Saban et al., including the number of doses received [22]. Vaccination against COVID-19 in Israel is available free of charge, but reduced accessibility of the low SES population to vaccination sites or to vaccine publicity and knowledge probably plays a role, necessitating public health-concentrated measures.

Our finding of reduced vaccination uptake by individuals with certain underlying comorbidities, which are actually associated with increased COVID-19-related complications and mortality, is very disturbing. In the multivariate analysis, we documented that smoking, asthma, and diabetes mellitus are significantly associated with low COVID-19 vaccination rates. Of note, individuals with obesity and with perceived other underlying medical conditions, such as cardiovascular, respiratory, and immune disorders, had high COVID-19 vaccine acceptance rates.

Multiple studies showed that smoking was associated with higher risks of increased burden of COVID-19 symptoms and COVID-related morbidity, including respiratory failure and death [23,24,25,26]. Furthermore, several large-scale meta-analyses have concluded that even a history of smoking is associated with a range of adverse outcomes including severe COVID-19 infection and mortality [27,28,29]. The low vaccine acceptance rates among smoking adults might reflect a tendency of this population not to adhere to medical recommendations and might be explained by the failure to administer the clear message that smoking is a risk factor for a severe course of COVID-19. A special effort is needed to clearly deliver this important knowledge.

Obesity has already been extensively reported as one of the chronic pre-existing conditions in adults who are at higher risk of severe COVID-19 disease, leading to hospitalization, admission to intensive care, and death [30,31,32,33,34]. Luckily, it seems that there is a high awareness among Israeli adults with obesity of the special need for COVID-19 vaccination, a very important factor in decreasing COVID-19-related morbidity and mortality.

The prevalence of asthma among COVID-19 patients varies greatly across countries and regions worldwide [35,36,37]. Several meta-analyses have investigated the association between pre-existing asthma and COVID-19 mortality worldwide [35,36,37], with inconsistent conclusions, possibly due to substantial variation in asthma severity and prevalence among different countries. Sunjaya et al. reported that COVID-19 patients with asthma had a significantly increased risk for mortality in Asia, but not in North America, South America and Europe [38]. Another recent study demonstrated that pre-existing asthma was significantly associated with a reduced risk for COVID-19 mortality in the United States [39]. The inconsistent data might have played a role in the significantly reduced vaccine acceptance among individuals with asthma, but their lower vaccination rate compared with the general population is unfortunate and requires focused attention.

Diabetes mellitus is one of the comorbidities closely related to the risk of morbidity and mortality in patients with COVID-19 [40,41,42]. Many studies showed that the proportion of diabetes is higher in COVID-19 patients with a severe clinical course and that people with diabetes are also more vulnerable to COVID-19 infection than those without diabetes [43,44]. Thus, the low vaccine acceptance rate in this population is very disturbing and is probably related mainly to unawareness of the severe course of COVID-19 in patients with diabetes mellitus.

Duan et al. [45] explored COVID-19 vaccination behavior and correlates in 645 diabetic patients from two hospitals in China between June and October 2021. They used multivariable logistic regression to determine predictors related to COVID-19 vaccine uptake. A total of 162 vaccinated and 483 unvaccinated eligible diabetic patients were included. Patients who believed that the COVID-19 syndrome is severe believed that vaccination can significantly reduce the risk of SARS-CoV-2 infection thought that vaccination is beneficial to themselves and others, and believed that relatives’ vaccination status has a positive impact on their vaccination behavior and were more likely to be vaccinated; worrying about the adverse health effects of COVID-19 vaccine was negatively correlated with the COVID-19 vaccination rate [45].

Benis et al. investigated COVID-19 vaccination adherence and hesitancy among 1644 US social media users, showing that these individuals have mostly a positive attitude towards COVID-19 vaccination and want to protect their families and their relatives as an act of civic responsibility [46]. Shimoni et al. conducted a cross-sectional survey, which found that seeking information from digital sources and non-health-related governmental agencies and family, friends, and influencers was associated with high vaccine intent [47]. These results strengthen our suggestion that using social media is an important means to increase vaccine uptake, especially when addressing targeted populations. Therefore, a special campaign to deliver information about the severe course of COVID-19 in patients with diabetes mellitus is suggested, for example, in forums and social media platforms directed to these patients.

The main strength of this study is the comprehensive and reliable data that were available on these study populations, including detailed demographics, underlying medical disorders, the medication used, and vaccine acceptance. Our study has several limitations. First, data were analyzed at a single district level and not at the nationwide population and, therefore, might not necessarily be representative of the entire population with potential bias. However, as shown above, the population of the district is diverse, with representation from the various ethnic and SES groups in the country. Second, we did not include in our analyses the fourth COVID-19 vaccine dose; however, this dose was recommended in Israel only for specific high-risk groups, thus vaccine acceptance rates were actually impossible to study. Third, it is always possible that additional variables that we could not study affected vaccine acceptance.

## 5. Conclusions

The main conclusion of the present study is that, although COVID-19 vaccine acceptance in the adult Israeli population is relatively high (>84%), it is significantly lower among certain populations, including in groups at high risk of severe course and complications of COVID-19, such as those with comorbidities. Targeted public health interventions aimed at these populations are required with consideration of their special social characteristics and underlying conditions. Targeted delivery of key messages to the identified populations is currently possible, for example, by community/religious leaders, newsletters, specialized media and social media networks. It is suggested that health policymakers use the aid of experts in online health communication and social networking. Targeted needs of special populations such as those with low SES should also be planned, for example, by making vaccination sites more accessible (mobile vaccination teams, availability after working hours, etc.).

## Figures and Tables

**Table 1:** Study group variables by COVID-19 vaccination.

Variable	Total	Vaccinated (≥1 Dose) *N* (%)	Unvaccinated *N* (%)	*p*-Value
Study cohort	246,543	207,911 (84%)	38,632 (16%)	
Age, mean ± SD	51.6 ± 19.7	53.2 ± 19.7	42.9 ± 17.3	
Age category, years 20–39	84,364	63,967 (75.6%)	20,667 (24.4%)	<0.001
40–59	71,433	60,576 (84.8%)	10,857 (15.2%)	
60–79	66,756	61,408 (92%)	5348 (8%)	
≥80	23,720	21,960 (92.6%)	1760 (7.4%)	
Gender				
Male	120,724	100,960 (83.6%)	19,764 (16.4%)	<0.001
Female	125,819	106,961 (85%)	18,868 (15%)	
Sector				
Non-ultra-Orthodox Jews	193,035	169,259 (87.7%)	23,766 (12.3%)	<0.001
Ultra-Orthodox Jews	37,511	25,771 (68.7%)	11,740 (31.3%)	
Arabs	15,997	12,881 (80.5%)	3116 (19.5%)	
Socioeconomic status				
Low	57,111	42,474 (74.4%)	14,637 (25.6%)	<0.001
Middle	96,624	81,138 (84%)	15,486 (16%)	
High	92,808	84,229 (90.8%)	8509 (9.2%)	
Previous influenza vaccination				
Vaccinated	101,429	95,834 (94.5%)	5595 (5.5%)	<0.001
Unvaccinated	145,114	112,077 (77.2%)	33,037 (22.8%)	

**Table 2:** Vaccination against COVID-19 by underlying medical conditions.

Underlying Conditions	Total	Vaccinated (≥1 Dose) *N* (%)	Unvaccinated *N* (%)	*p*-Value
Obesity (BMI > 30)	56,753	49,993 (88.1%)	6760 (11.9%)	<0.001
Diabetes mellitus	34,877	31,722 (91%)	3155 (9%)	<0.001
Asthma	19,434	16,594 (85.4%)	2840 (14.6%)	<0.001
COPD	6619	5996 (90.6%)	623 (9.4%)	<0.001
Cystic fibrosis	43	41 (95.3%)	2 (4.7%)	0.0754
Cirrhosis	707	621 (87.8%)	86 (12.2%)	0.0119
Smoker	65,410	54,092 (82.7%)	11,318 (17.3%)	<0.001
Former smoker	48,134	42,245 (87.8%)	5889 (12.2%)	<0.001
Cardiac disease ^1^	25,065	23,164 (92.4%)	1901 (7.6%)	<0.001
Hypertension	55,685	51,516 (92.5%)	4169 (7.5%)	<0.001
CVA	10,803	9851 (91.2%)	952 (8.8%)	<0.001
Malignancy ^2^	22,772	20,965 (92.1%)	1807 (7.9%)	<0.001
Chronic renal failure	7869	7136 (90.7%)	733 (9.3%)	<0.001
Solid organ transplantation	8344	7900 (94.7%)	444 (5.3%)	<0.001
Bone marrow transplantation	318	279 (87.7%)	39 (12.3%)	0.1108
Down syndrome	1681	1466 (87.2%)	215 (12.8%)	0.0013
Hematologic diseases ^3^	57	51 (89.5%)	6 (10.5%)	0.376
Neurologic diseases ^4^	7753	7102 (91.6%)	651 (8.4%)	<0.001
Depression	18,813	16,466 (87.5%)	2347 (12.5%)	<0.001
Rheumatologic diseases ^5^	4799	4323 (90.1%)	476 (9.9%)	<0.001
Biological therapy ^6^	1036	962 (92.9%)	74 (7.1%)	<0.001
Steroid therapy ^7^	485	439 (90.5%)	46 (9.5%)	<0.001

BMI, body mass index; COPD, chronic obstructive pulmonary disease; CVA, cerebrovascular accident. ^1^ Cardiac diseases: including ischemic heart disease, congestive heart failure, and cardiomyopathy. ^2^ Malignancy: patients treated due to malignancy in the previous five years, including patients with multiple myeloma. ^3^ Hematologic diseases: including thalassemia major and sickle cell anemia. ^4^ Neurologic diseases: including Alzheimer’s disease, dementia, Parkinson’s disease, and multiple sclerosis. ^5^ Rheumatologic diseases: including psoriatic arthritis, systemic lupus erythematosus, rheumatoid arthritis, scleroderma (systemic sclerosis), and dermatomyositis.  ^6^ Biological therapy: including alemtuzumab, adalimumab, certolizumab, infliximab, etanercept, rituximab, and anakinra.  ^7^ Steroid therapy: including prednisone or prednisolone at a dosage of ≥20 mg/day for at least one week.

**Table 3:** Characteristics of study group by the number of COVID-19 vaccine doses received.

Variable	Total	No. of Vaccinations Received			
		One Dose *N* (%)	Two Doses *N* (%)	Three Doses *N* (%)	*p*-Value
Study cohort	207,911	15,715 (7.5%)	34,242 (16.5%)	157,954 (76%)	<0.001
Age, mean ± SD	44.0 ± 19.9	36.0 ± 18.6	36.1 ± 18.3	46.5 ± 19.7	
Age category, years					
20–39	63,967	7547 (11.8%)	16,183 (25.3%)	40,237 (62.9%)	<0.001
40–59	60,576	4863 (8%)	10,759 (17.8%)	44,954 (74.2%)	
60–79	61,408	2170 (3.5%)	5178 (8.4%)	54,060 (88%)	
≥80	21,960	1135 (5.2%)	2122 (9.7%)	18,703 (85.1%)	
Gender					
Male	100,960	7352 (7.3%)	16,893 (16.7%)	76,715 (76%)	0.0098
Female	106,951	8363 (7.8%)	17,349 (16.2%)	81,239 (76%)	
Sector					
Non-ultra-Orthodox Jews	169,259	8939 (5.3%)	23,528 (13.9%)	136,792 (80.8%)	<0.001
Ultra-Orthodox Jews	25,771	4730 (18.4%)	6413 (24.9%)	14,628 (56.7%)	
Arabs	12,881	2046 (15.9%)	4301 (33.4%)	6534 (50.7%)	
Socioeconomic status					
Low	42,474	6036 (14.2%)	9867 (23.2%)	26,571 (62.6%)	<0.001
Middle	81,138	6484 (8%)	15,137 (18.7%)	59,517 (73.3%)	
High	84,299	3195 (3.8%)	9238 (11%)	71,866 (85.2%)	
Previous influenza vaccination					
Vaccinated	95,836	4359 (4.5%)	9662 (10.1%)	81,813 (85.4%)	<0.001
Unvaccinated	112,077	11,356 (10.1%)	24,580 (21.9%)	76,141 (68%)	

**Table 4:** Characteristics of study group with underlying medical conditions by the number of COVID-19 vaccine doses received.

Underlying Conditions	Total	Received Only One Vaccine Dose *N* (%)	Received Two Vaccine Doses *N* (%)	Received Three Vaccine Doses *N* (%)	*p*-Value
Obesity (BMI > 30)	49,993	3742 (7.5%)	7020 (14%)	39,231 (78.5%)	<0.001
Diabetes mellitus	31,722	1761 (5.5%)	3704 (11.7%)	26,257 (82.8%)	<0.001
Asthma	16,594	1247 (7.5%)	2728 (16.4%)	12,619 (76.1%)	0.966
COPD	5996	312 (5.2%)	723 (12.1%)	4961 (82.7%)	<0.001
Cystic fibrosis	41	4 (9.8%)	4 (9.8%)	33 (80.5%)	<0.001
Cirrhosis	621	45 (7.2%)	80 (12.8%)	496 (80%)	<0.001
Smoker	54,092	3120 (5.8%)	9944 (18.4%)	41,028 (75.8%)	<0.001
Former smoker	42,245	2126 (5%)	5648 (13.4%)	34,471 (81.6%)	<0.001
Cardiac disease ^1^	23,164	1161 (5%)	2450 (10.6%)	19,553 (84.4%)	<0.001
Hypertension	51,516	2408 (4.7%)	5001 (9.7%)	44,107 (85.6%)	<0.001
CVA	9851	636 (6.5%)	1241 (12.6%)	7974 (80.9%)	<0.001
Malignancy ^2^	20,965	906 (4.3%)	2111 (10.1%)	17,948 (85.6%)	<0.001
Chronic renal failure	7136	442 (6.2%)	951 (13.3%)	5743 (80.5%)	<0.001
Solid organ transplantation	7900	287 (3.6%)	687 (8.7%)	6926 (87.7)	<0.001
Bone marrow transplantation	279	13 (4.7%)	32 (11.4%)	234 (83.9%)	0.0081
Down syndrome	1466	92 (6.3%)	244 (16.6%)	1130 (77.1%)	<0.001
Hematologic diseases ^3^	51	7 (13.7%)	11 (21.6%)	33 (64.7%)	<0.001
Neurologic diseases ^4^	7102	598 (8.4%)	1069 (15.1%)	5435 (76.5%)	<0.001
Depression	16,466	965 (5.9%)	2270 (13.8%)	13,231 (80.3%)	<0.001
Rheumatologic diseases ^5^	4323	205 (4.7%)	519 (12%)	3599 (83.3%)	<0.001
Biological therapy ^6^	962	45 (4.7%)	107 (11.1%)	810 (84.2%)	<0.001
Steroid therapy ^7^	145	23 (15.9%)	27 (18.6%)	95 (65.5%)	<0.001

BMI, body mass index; COPD, chronic obstructive pulmonary disease; CVA, cerebrovascular accident. ^1^ Cardiac diseases: including ischemic heart disease, congestive heart failure, and cardiomyopathy. ^2^ Malignancy: patients treated due to malignancy in the previous five years, including patients with multiple myeloma. ^3^ Hematologic diseases: including thalassemia major and sickle cell anemia. ^4^ Neurologic diseases: including Alzheimer’s disease, dementia, Parkinson’s disease, and multiple sclerosis. ^5^ Rheumatologic diseases: including psoriatic arthritis, systemic lupus erythematosus, rheumatoid arthritis, scleroderma (systemic sclerosis), and dermatomyositis. ^6^ Biological therapy: including alemtuzumab, adalimumab, certolizumab, infliximab, etanercept, rituximab, and anakinra. ^7^ Steroid therapy: including prednisone or prednisolone at a dosage of ≥20 mg/day for at least one week.

**Table 5:** Multivariate analysis of variables associated with COVID-19 vaccination rates.

Variable	Odds Ratio	Lower 95% CI	Upper 95% CI	*p*-Value
Age	1.02	1.019	1.022	<0.001
Female	0.842	0.791	0.895	<0.001
Arab	0.453	0.394	0.52	<0.001
Arab female	0.728	0.611	0.868	<0.001
Ultra-Orthodox Jews	0.484	0.438	0.536	<0.001
Ultra-Orthodox female	1.136	1.007	1.282	0.038
Low SES	1.418	1.363	1.475	<0.001
Previous influenza vaccination	3.88	3.663	4.11	<0.001
Smoking	0.901	0.85	0.955	<0.001
Asthma	0.843	0.782	0.909	<0.001
Diabetes mellitus	0.926	0.873	0.983	0.011
Obesity	1.086	1.029	1.145	0.003

SES, socioeconomic status.

## Data Availability

Data sources of the participants are not publicly available.

## Abbreviations

COVID-19—coronavirus disease 2019; SARS-CoV-2—severe acute respiratory syndrome coronavirus 2; WHO—World Health Organization; CHS—Clalit Health Services; SES—socioeconomic status; DM2–diabetes mellitus type 2; CI—confidence interval.
